# Electric Fields Are a Key Determinant of Carbapenemase Activity in Class A β-Lactamases

**DOI:** 10.1021/acscatal.3c05302

**Published:** 2024-04-23

**Authors:** Hira Jabeen, Michael Beer, James Spencer, Marc W. van der Kamp, H. Adrian Bunzel, Adrian J. Mulholland

**Affiliations:** † Centre for Computational Chemistry, School of Chemistry, University of Bristol, BS8 1TS Bristol, United Kingdom; ‡ School of Cellular and Molecular Medicine, University of Bristol, BS8 1TD Bristol, United Kingdom; § Department of Biosystem Science and Engineering, ETH Zurich, 4056 Basel, Switzerland; ∥ School of Biochemistry, University of Bristol, BS8 1TD Bristol, United Kingdom

**Keywords:** electric fields, antimicrobial resistance, biocatalysis, β-lactamases, quantum mechanics/molecular mechanics

## Abstract

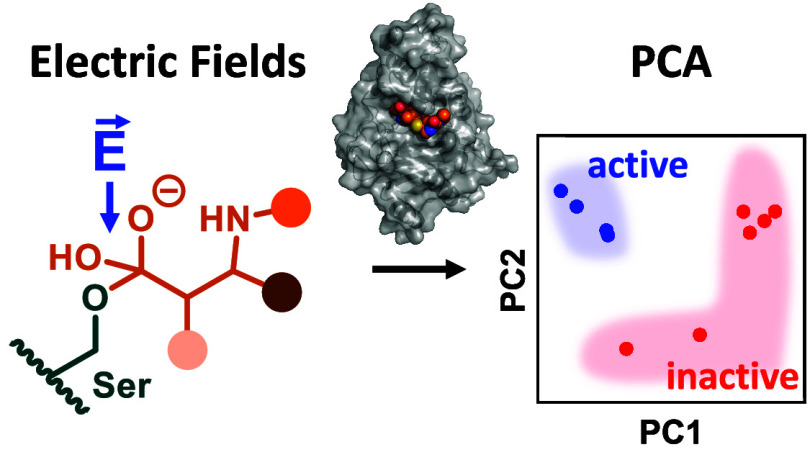

Resistance to antibiotics is a public health crisis. Although carbapenems are less susceptible to resistance than other β-lactam antibiotics, β-lactamases mediating resistance against these drugs are spreading. Here, we dissect the contributions of electric fields to carbapenemase activity in class A β-lactamases. We perform QM/MM molecular dynamics simulations of meropenem acyl-enzyme hydrolysis that correctly discriminate carbapenemases. Electric field analysis shows that active-site fields in the deacylation transition state and tetrahedral intermediate are important determinants of activity. The active-site fields identify several residues, some distal, that distinguish efficient carbapenemases. Our field analysis script (www.github.com/bunzela/FieldTools) may help in understanding and combating antibiotic resistance.

Antimicrobial resistance is an escalating global crisis, threatening human lives and many aspects of modern medicine. Around 1.2 million deaths annually are a direct result of infections with resistant pathogens.^1^ The overuse of antibiotics compounds this crisis and accelerates the evolution of antimicrobial resistance.^2,3^ Alarmingly, resistance development is outpacing the discovery of new antibiotics.^4,5^ Thus, as sequence information becomes more widely available as part of clinical microbiology workflows, there is an urgent need for reliable tools to predict the activity spectrum of emerging resistance genes and guide the design of next-generation antibiotics.^2^

In Gram-negative bacteria such as *Escherichia coli*, resistance to β-lactams, the most commonly prescribed antibiotic class, arises primarily through antibiotic hydrolysis by β-lactamases.^6−8^ In class A β-lactamases, hydrolysis follows a two-step mechanism (Figures 1a and S1).^9^ β-Lactam breakdown commences with a nucleophilic attack of a catalytic serine on the amide carbonyl, leading to the formation of a covalent acyl-enzyme complex (AE) via a tetrahedral intermediate. Subsequently, these enzymes use a glutamate base to deprotonate a deacylating water and hydrolyze the AE complex via a second tetrahedral intermediate (TI). Hydrolysis of the AE intermediate is often slower than its formation,^10−12^ although other steps may also be rate-limiting.^13^

**Figure 1:**
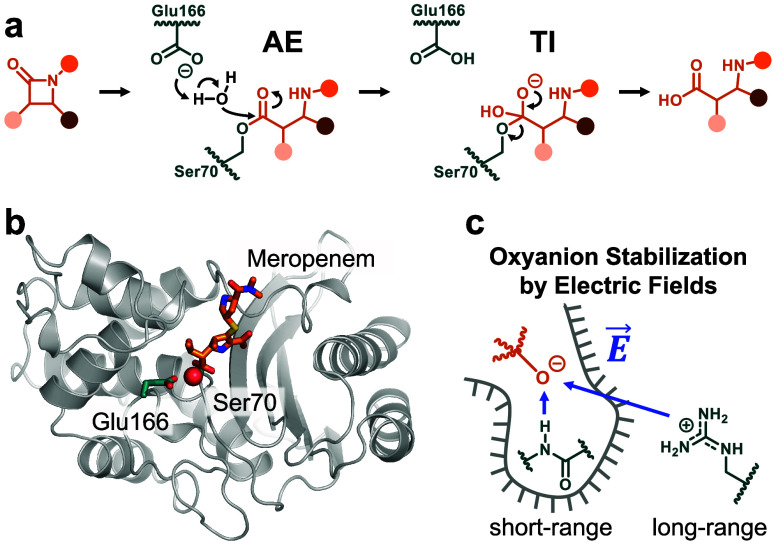
Electric fields and β-lactamase activity. (a) β-Lactam cleavage in class A β-lactamases involves a covalent acyl-enzyme intermediate (AE) that is hydrolyzed via a tetrahedral intermediate (TI). (b) TEM-116:meropenem AE complex (catalytic Glu166 and Ser70, teal; meropenem, orange). (c) Electrostatic stabilization of the oxyanion intermediate is key to activity in β-lactamases, but identifying the residues involved can be challenging.

Various β-lactams, such as the carbapenem meropenem, have been specifically engineered to result in slow TI hydrolysis to inhibit β-lactamase activity and prevent resistance.^14−17^ Nevertheless, clinically relevant class A β-lactamases, such as *Klebsiella pneumoniae* carbapenemase (KPC), have emerged that efficiently break down meropenem to confer resistance.^17−23^ Understanding the molecular origins of efficient AE hydrolysis is crucial to elucidating β-lactamase activity and resistance phenotypes.

Electrostatic interactions play a pivotal role in enzyme catalysis,^24−26^ and electrostatic stabilization of transient oxyanionic species such as the β-lactamase deacylation TI is key to activity in many hydrolytic enzymes.^27−29^ We hypothesized that residues critical for β-lactamase activity could be readily identified from their electrostatic interactions with the TI oxyanion. Due to the long-range nature of electrostatic interactions, the identified residues might even involve positions far away from the active site. Thus, electrostatic analysis might help identify catalytically relevant remote residues, which is a major challenge for understanding existing biocatalysts and designing novel enzymes.^30,31^

Electric fields, experimentally observable from vibrational spectroscopy, represent a physical metric that can be used to quantify electrostatic effects.^24−26^ Here, we set out to perform atomistic molecular dynamics (MD) simulations to compare electric field effects in four class A β-lactamases with high hydrolytic activity toward the carbapenem meropenem (carbapenemases: KPC-2, NMC-A, SFC-1, and SME-1)^17−23,65−69^ with those in six enzymes with insufficient activity to confer resistance (non-carbapenemases: BlaC, CTX-M-16, SHV-1, TEM-1, TEM-52, and TEM-116).^14,16,15,17,68−72^ To that end, AE deacylation was simulated by hybrid quantum mechanics/molecular mechanics (QM/MM) MD using DFTB2/ff14SB (Figure S2a).^32−36^ As benchmarked in our previous work,^10,11^ 2D umbrella sampling was used to simulate AE hydrolysis by following the deprotonation of the deacylating water and its nucleophilic attack upon the acyl-enzyme carbonyl. In addition, deacylation was simulated using the adaptive string method.^32^ The string method works by projecting all of the collective variables onto a single reaction coordinate. This allows the calculations to be focused on the minimum free energy path and increases sampling efficiency compared with conventional umbrella sampling.

Both QM/MM sampling approaches give barriers (Δ*G*^‡^_calc_) that correlate well with experimental activity (Δ*G*^‡^_exp_; string method, *R*^2^ = 0.82; 2D umbrella sampling, *R*^2^ = 0.67; Figures 2, S3, and S4). The calculated barriers are generally lower than the experimental activation energies (Table S1) because of limitations of the DFTB2 method, as noted previously.^10,11^ The string method gave lower barriers than those from 2D umbrella sampling. By its nature, the string method allows for better definition and more comprehensive sampling of the minimum free energy path.^32^ Sampling is further enhanced using replica exchange in the adaptive string method implementation,^32^ which we did not perform during 2D umbrella sampling. Combined, these factors likely decreased the calculated barriers and improved the correlation of Δ*G*^‡^_calc_ with Δ*G*^‡^_exp_ for the sting method compared to 2D umbrella sampling. Nonetheless, both methods provide barriers that agree with Δ*G*^‡^_exp_ suggesting that the MD trajectories from both approaches are suitable to assess electrostatic effects promoting AE hydrolysis.

**Figure 2:**
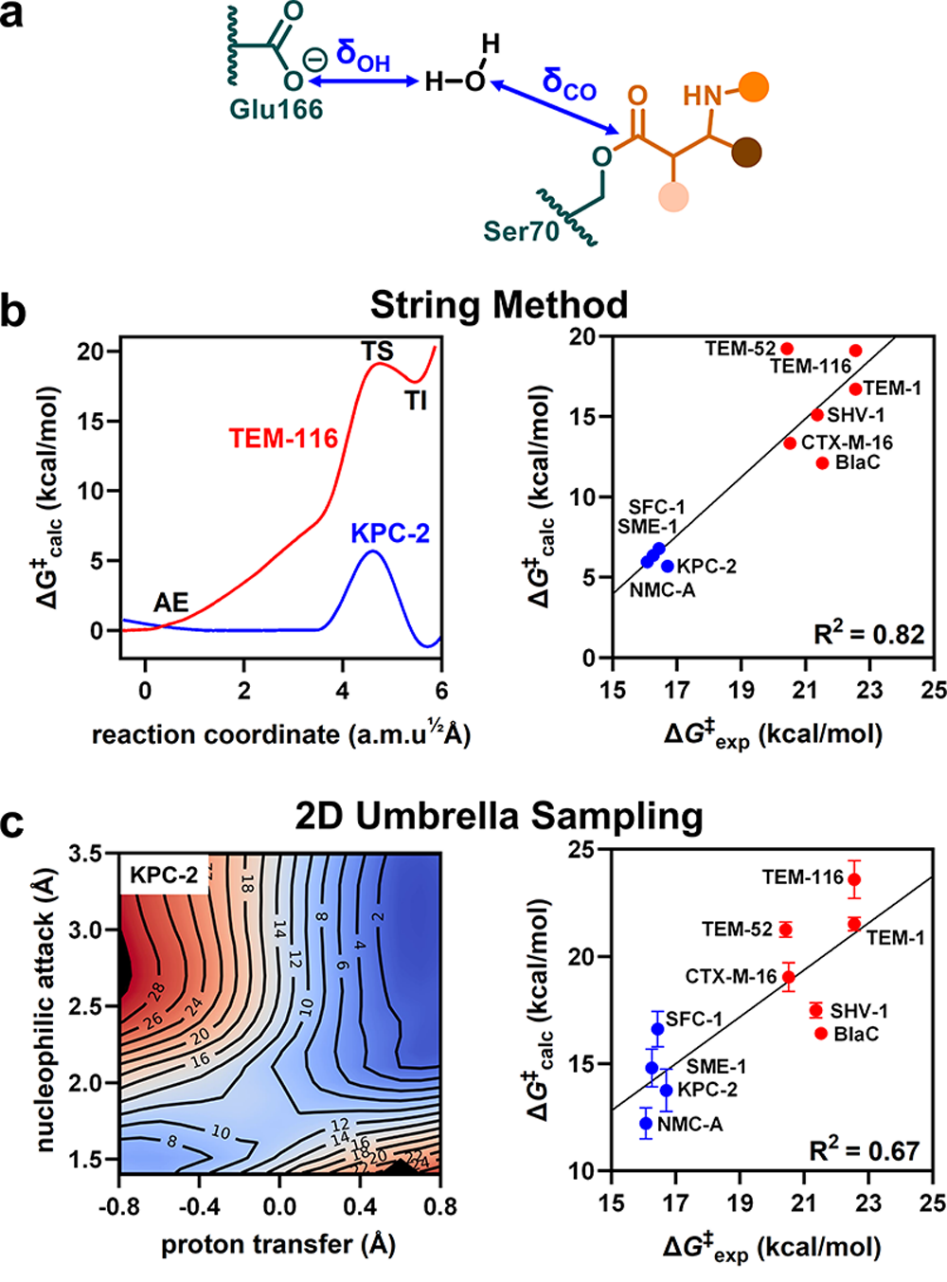
Calculated free energy barriers for AE deacylation and experimental *k*_cat_ values. (a) Reaction coordinates for AE hydrolysis comprise the proton transfer (δ_OH_) and nucleophilic attack (δ_CO_). (b and c) Δ*G*^‡^_calc_ from the string method (b) and 2D umbrella sampling (c) correlate well with Δ*G*^‡^_exp_ (see Table S1 for experimental values; blue, carbapenemases; red, non-carbapenemases; for 2D umbrella sampling, proton transfer is defined as the antisymmetric combination of the base and water O–H distances; error bars represent the standard error of 10 independent calculations).

To investigate the electrostatic stabilization of the negative charge accumulating on the TI carbonyl oxygen, we determined electric fields along the β-lactam C=O bond. Fields in the AE, TS, and TI states were determined from the obtained QM/MM trajectories. Electric field vectors *E⃗* projected by the enzyme onto the C=O bond were calculated based on the point charges in the topology file (eq S1). *E⃗* was subsequently projected onto the C=O bond (eq S2) to give rise to the effective field vector (*E⃗*_eff_) stabilizing the C=O dipole. To quantify electric field effects, the analysis presented here focuses on the magnitude of the field vector *E*_eff_ (eq S3, Figure 3b). The FieldTools script for electric field calculation is available at www.github.com/bunzela/FieldTools. To mask effects intrinsic to the reaction, the “reactive” part of the substrate, comprising the C=O bond with its adjacent carbon atoms and the deacylating water, was excluded from the *E*_eff_ calculations (Figure S2b). Finally, we note that, while we focus on the results from 2D umbrella sampling are qualitatively similar, underscoring the significance of our findings (Figures S5, S8 and S9).

**Figure 3:**
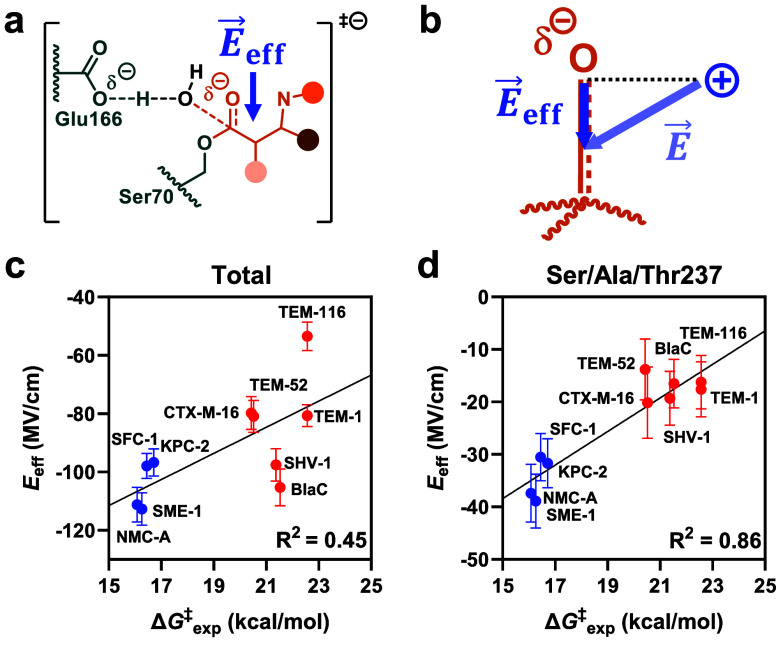
Electric fields in carbapenemases and non-carbapenemases. (a) *E*_eff_ was calculated in the TS from (b) the effective electric field *E⃗*_eff_ projected by the enzyme along the carbonyl bond of the carbapenem oxyanion (orange). (c) While the total *E*_eff_ in the TS ensemble only weakly correlates with activity, (d) the *E*_eff_ contributions of individual positions that interact with the oxyanion agree well with Δ*G*^‡^_exp_ (*E*_eff_ values based on the string method; see Figure S8 for *E*_eff_ values based on 2D umbrella sampling and other per-residue values; error bars reflect the standard error of 10 independent replicas).

The total *E*_eff_ was always negative and increased in magnitude from the AE to the TS and TI ensembles (Figures 3 and S5, Table S2). This *E*_eff_ increase indicates that the protein reorganizes during the reaction to better accommodate the oxyanion in the TS and TI. The *E*_eff_ increase is particularly pronounced for highly active variants, leading to much better TI stabilization in the carbapenemases than in the non-carbapenemases. Overall, *E*_eff_ values ranged between −53 and −115 MV/cm in the TS ensemble, which agrees well with the fields observed in other enzymes that utilize oxyanion stabilization for catalysis.^25−29^

To understand how the electric fields affect activity, we analyzed how *E*_eff_ changed with Δ*G*^‡^_exp_. As expected, more active carbapenemases displayed more negative fields, which we quantify from the slope determined from the *E*_eff_ vs Δ*G*^‡^_exp_ correlations. These slopes revealed that the TS and TI ensembles showed a much larger difference in *E*_eff_ between enzymes than the AE state (4.5, 4.5, and 1.8 (MV/cm)/(kcal/mol)). Note that the TI is often treated as a TS analogue because it is more defined, and thus experimentally and computationally more accessible, than the TS. Our work confirms that the TI and TS have similar electrostatic properties. Nonetheless, marginally better correlation coefficients with Δ*G*^‡^_exp_ were observed for the TS (Figure S5). The TS is thus the more accurate state for studying the reaction barrier, highlighting the importance of analyzing transition states to understand catalysis.

Overall, the *E*_eff_ analysis reproduced the expected electrostatic oxyanion stabilization that is vital to β-lactamase activity. Nonetheless, *E*_eff_ reflects only one of several catalytic contributions, which might explain outliers in the correlation of *E*_eff_ with Δ*G*^‡^_exp_ (Figures 3 and S5). Organization of the active site through extended hydrogen-bonding networks (Figure S6a), water-mediated substrate tautomerization, and a disulfide bond between Cys69 and Cys238 have all previously been shown to contribute to carbapenemase activity.^12,19,37−40^ Furthermore, BlaC contains an Asn132Gly mutation, which precludes a hydrogen bond with the carbapenem 6α-hydroxyethyl group and decreases substrate preorganization (Figure S6b).^41^ We note that experimental deacylation rate constants are only available for the reaction of meropenem with TEM-52 and BlaC.^14,15^ For the other β-lactamases, *k*_cat_ values were used to approximate the barrier (see discussion in Figure S1a). Although deacylation does not always limit *k*_cat_, carbapenemases are generally distinguished by their ability to deacylate rapidly, which is related to a strong electric field stabilizing the TS oxyanion.

FieldTools can readily partition the total *E*_eff_ into individual contributions to identify parts of the system that provide most of the electrostatic effect. Per-residue *E*_eff_ values were thus calculated to identify which protein residues drive the change in *E*_eff_ between the studied β-lactamases. Because the β-lactamases differ in size, a structure-based sequence alignment was performed, and loops with varying lengths were removed from the analysis (residues 26–28, 50–56, 85–90, 141–144, 240–241, and 268–274, Figure S7). For reference, our analysis uses the residue numbers of the TEM variants. Substantial changes in Eeff with Δ*G*^‡^_exp_ ranging from −2.6 to +3.2 (MV/cm)/(kcal/mol) were observed for several residues. Large *E*_eff_ changes were observed both for residues known to be involved in electrostatic oxyanion stabilization, such as the oxyanion-hole donating residue 237 (Figure 3d), as well as for nonobvious remote residues up to 15 Å away from the oxyanion (Figure S8).

In addition to the protein, water can substantially impact biocatalysis.^24,42,43^ Separating the solvent and protein *E*_eff_ revealed that the change in total *E*_eff_ between variants is dominated by the protein (−117 to 63 MV/cm), with solvent fields ranging between −6 and 18 MV/cm (Figure S5b). While the change of solvent *E*_eff_ with Δ*G*^‡^_exp_ (1.4 (MV/cm)/(kcal/mol)) was less pronounced than that of individual protein residues, it is feasible that changes in active-site solvation contribute to the carbapenemase activity differences.

The calculated per-residue *E*_eff_ values were subjected to principal component analysis (PCA) to identify critical sites modulating the electrical field between enzymes (Figure S9). PCA is a statistical method that can reveal trends in complex data sets by projecting data onto a smaller set of principal components. The landscape of the first and second principal components (PC1 and PC2, representing 54% and 16% of total variance) revealed a defined cluster for the carbapenemases, while non-carbapenemases populated a broad but distinct distribution. PCA of per-residue *E*_eff_ values thus clearly distinguishes carbapenemases from non-carbapenemases and suggests that high β-lactamase activity requires a distinct electrostatic configuration (Figure 4a).

**Figure 4:**
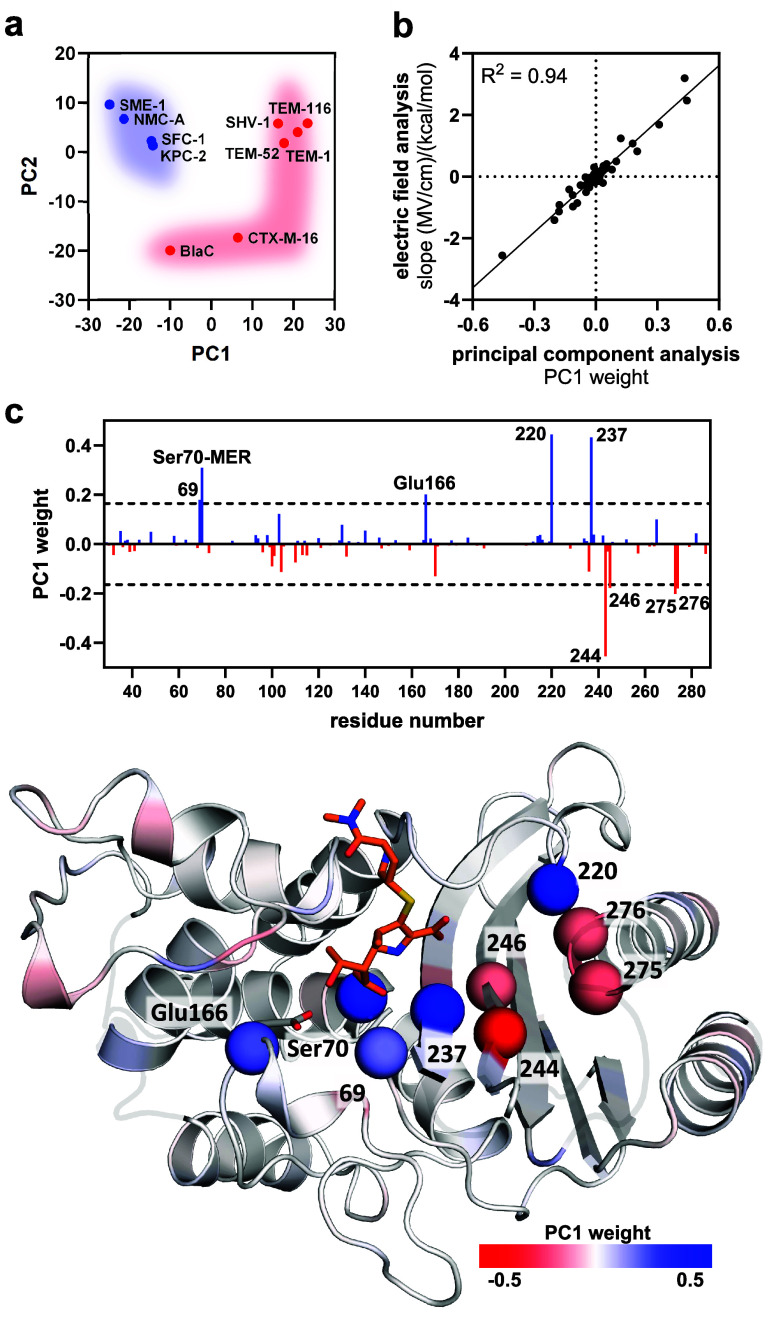
Contribution of individual residues to the electric field. (a) PCA of the per residue *E*_eff_ distinguishes carbapenemases (blue) from non-carbapenemases (red, shaded areas added for illustration). (b) The analysis of *E*_eff_ vs Δ*G*^‡^_exp_ agrees with the PCA. (c) PC1 weights reveal that a cluster of seven residues and the catalytic residues (spheres) dominates the difference in *E*_eff_ (blue, beneficial; red, detrimental; Ser70-MER, field effect of the meropenem acylated Ser70; meropenem, orange; dashed line, cutoff; data based on string method; see Figure S9 for results based on 2D umbrella sampling).

Principal component weights can provide detailed insights into which parameter, here, which residue position, has the strongest effect in a data set. We hypothesized that PC1 weights could reveal “critical” positions that dominate the *E*_eff_ change between variants. The weights of the two catalytic and seven other residues differed by ≥2.5 standard deviations from the average (Figure 4c, Table 1). Comparison of the weights with the *E*_eff_ vs Δ*G*^‡^_exp_ slopes shows that positive weights correspond to residues that exert catalytically more beneficial fields as activity increases (Figures 4b and S9). These comprise the catalytic base Glu166, the acylated Ser70, and residue 237, whose backbone amide formes the oxyanion hole together with Ser70. Several other residues, some located up to 15 Å away from the oxyanion, also had pronounced weights. Residues 69 and 220 had positive weights, while residues 244, 246, 275, and 276 had negative weights.

**Table 1:** Residues Distinguishing Carbapenemases Activity^a^

variant	69	220	237	244	246	275	276
BlaC	C	R	T	A	D	R	E
CTX-M-16	C	S	S	T	D	R	R
SHV-1	M	L	A	R	I	R	N
TEM-1	M	L	A	R	I	R	N
TEM-52	M	L	A	R	I	R	N
TEM-116	M	L	A	R	I	R	N
KPC-2	C	R	T	A	D	S	E
NMC-A	C	R	S	A	D	E	D
SFC-1	C	R	T	A	D	S	D
SME-1	C	R	S	A	D	S	D

a Identfied by PCA of per-residue electric fields (Figure 4c).

Notably, the per-residue weights excellently agreed with the slopes determined from the *E*_eff_ vs Δ*G*^‡^_exp_ analysis, which strongly supports that the PCA captures catalytically relevant effects (Figures 4b and S9). We emphasize that neither Δ*G*^‡^_calc_ nor Δ*G*^‡^_exp_ were included in the PCA. The PCA is thus not biased by any activity data and solely reflects changes in the active-site electrostatics between variants. Nonetheless, both the PCA and the per-residue correlations of *E*_eff_ with Δ*G*^‡^_exp_ identified similar residues with substantial electrostatic contributions (Figures 4b and S9). Electric field analysis revealed that *E*_eff_ is dominated by the catalytic residues and a cluster of seven residues around the oxyanion hole (Figure 4c), with PCA reliably pinpointing these catalytically relevant and partially nonobvious residues.

All carbapenemases studied here have an arginine residue at position 220, while the non-carbapenemases all have at least one arginine at position 244, 275, or 276 (Figure S10). Notably, the only non-carbapenemase with Arg220 is BlaC, which also shows an unexpectedly strong overall *E*_eff_ as discussed above (Figure 3c). Previously, mutagenesis studies of class A carbapenemases revealed that introducing charged residues at several of our identified positions substantially affects activity.^44−50^ For instance, the R244A mutation in TEM-1 impairs hydrolysis of various penicillin and cephalosporin-based β-lactams, but the introduction of arginine at position 220, 272, or 276 can partially recover activity.^44^ Although some positions are known, understanding and predicting the effect of mutations at these positions have remained challenging. Our electric field analysis provides a quantitative description of the mutational effects. Per-residue *E*_eff_ values show that an arginine at position 220 is catalytically superior for meropenem hydrolysis compared to position 244, 275, or 276 (Figure S8a). Our analysis shows how the *E*_eff_ of a residue depends strongly on its position within the protein. Thus, the change in the *E*_eff_ of residues such as arginine at varying positions is probably an important discriminator for carbapenemase activity.

In summary, combining electric field calculations with principal component analysis enabled a detailed per-residue study of the electrostatic determinants of β-lactamase activity. Our analysis revealed a cluster of seven residues that dominates electrostatic oxyanion stabilization and, together with the two established catalytic residues, differentiates carbapenemases from non-carbapenemases. Given that resistance genes in pathogenic strains from patient samples can now be reliably identified by genome sequencing,^2^ we anticipate that electric field calculations may aid in predicting the resistance spectrum of emerging enzymes. In that regard, our assay might be substantially accelerated by simulating the metastable TI alone to be time-compatible with rapid sequencing and to provide clinical guidance.

Our work underscores the potential of integrating reaction simulations and electric field calculations to discern effects along the reaction coordinate.^51−57^ Simulation offers various advantages compared to experimental methods that typically rely on vibrational probes to determine electric fields: (1) computational assays are not limited to transition state analogs or ground state substrates; (2) specific states along the catalytic cycle can readily be studied; and (3) electric fields can easily be partitioned into individual components.

Electric field analysis provides an accessible framework for studying enzyme reorganization during reaction, as exemplified here by the increasing electric fields from AE to TS and TI. The data provided by FieldTools could feasibly inform detailed PCA or cross-correlation analysis of *E*_eff_ between states or within a given state to provide a much deeper understanding of the electrostatic environment and its relation to catalysis.

Dissecting per-residue electrostatic effects by PCA was instrumental in pinpointing residues that distinguish carbapenemase activity. Notably, PCA allowed determining effects without biasing the analysis with reaction barriers; the analysis solely reflects the most pronounced difference in the electrostatics between variants. Nonetheless, PCA reliably reproduced the catalytically important residues found by comparing *E*_eff_ with Δ*G*^‡^_exp_, underscoring the catalytic relevance of the results.

Electrostatic catalysis and electric fields have gained considerable attention in enzyme design and engineering, for instance, in tailoring active-site electrostatics^58−62^ and introducing remote catalytic interactions.^30,31^ To be useful for enzyme design, computational analyses must capture catalytic interactions with speeds compatible with the state-of-the-art design algorithms. Electric field analysis can achieve this speed. In addition, total or residue-based *E*_eff_ values could be used as features in machine learning models aimed at understanding catalytic activity in existing enzymes and engineering novel biocatalysts.^63,64^

In conclusion, our work shows that highly optimized electric fields in naturally evolved β-lactamases can give rise to antibiotic resistance. Therein, our FieldTools script provides an efficient means of dissecting electrostatic catalysis. Electric field analysis can identify molecular determinants of antibiotic resistance, which might help to forecast the evolution of resistance and inform the design of next-generation antibiotics.

## Data Availability

The FieldTools electric field calculation script is available at www.github.com/bunzela/FieldTools and the University of Bristol data repository, at https://doi.org/10.5523/bris.1wk7t08aubwq722s7fs9l5lw8u. The latter furthermore hosts all input files required to reproduce the MD simulations, as well as the MD trajectories generated during this work.
