# Supplementary material for: Electric Fields Are a Key Determinant of Carbapenemase Activity in Class A β-Lactamases
Source: ACS Catal. 2024 Apr 23;14(9):7166–72. doi: 10.1021/acscatal.3c05302 (PMC11075022; doi:10.1021/acscatal.3c05302)
Supplement: Supplementary file 1 — cs3c05302_si_001.pdf [file cs3c05302_si_001.pdf]

Supporting information for:

Electric Fields Are a Key Determinant of Carbapenemase Activity in class A β-lactamases

Hira Jabeen,[a] Michael Beer,[b] James Spencer,[b] Marc W. van der Kamp,[a,d] H. Adrian Bunzel,[a,c,*] and Adrian J. Mulholland[a,*]

[a] *Centre for Computational Chemistry, School of Chemistry, University of Bristol, BS8 1TS Bristol, UK*

[b] *School of Cellular and Molecular Medicine, University of Bristol, BS8 1TD Bristol, UK*

[c] *Department of Biosystem Science and Engineering, ETH Zurich, 4056 Basel Switzerland*

[d] *School of Biochemistry, University of Bristol, BS8 1TD Bristol, UK*

[*] *Corresponding authors: adrian.bunzel@bsse.ethz.ch, adrian.mulholland@bristol.ac.uk*

Table of Contents

1. Methods 2
   1.1. System setup 2
   1.2. String method free energy calculations 3
   1.3. 2D umbrella sampling free energy calculations 4
   1.4. Electric field calculations 5
2. Supporting Tables 6
   Tab. S1 | Free energy barriers for meropenem hydrolysis. 6
   Tab. S2 | Calculated electric fields. 6
3. Supporting Figures 7
   Fig. S1 | Carbapenemase mechanism. 7
   Fig. S2 | QM region and Field calculations. 8
   Fig. S3 | Free energy barriers from string calculations. 9
   Fig. S4 | Free energy barriers from 2D umbrella sampling. 10
   Fig. S5 | Electric fields decomposition into individual states. 11
   Fig. S6 | Active site interactions in carbapenemases and non-carbapenemases. 12
   Fig. S7 | Structure-based sequence alignment. 13
   Fig. S8 | Per-residue electric fields. 14
   Fig. S9 | PCA reveals residues key to electrostatic catalysis. 15
   Fig. S10 | Residues distinguishing carbapenemases from non-carbapenemases 16
4. References 17

1. Methods

MD simulations were based on previously built systems and generated parameters (see 2.1 System setup), and the 2D Umbrella sampling (see 2.3 2D umbrella sampling free energy calculations) were run similarly to tested and benchmarked methods.[1,2] Where simulations were available,[1] starting structures were taken from 300 ps QM/MM simulations from this previous work.

1.1. System setup

We studied ten class A β-lactamases selected for their clinical relevance and capacity to either hydrolyze carbapenems (KPC-2, NMC-A, SFC-1, and SME-1),15‑21 or that were inhibited by carbapenems (BlaC, CTX-M-16, SHV-1, TEM-1, TEM-52, and TEM-116).[3-6] Acyl-enzyme (AE) models were based on X-ray crystal structures with the following PDB IDs: 3DWZ, 1YLW, 2ZD8, 1M40, 1HTZ, 1BT5, 2OV5, 1BUE, 4EV4, and 1DY6.[7-16] For BlaC and SHV-1, meropenem-bound acyl-enzyme crystal structures were available. The TEM-1 crystal structure was obtained in complex with a boronic acid transition state analog, which was replaced by meropenem using PyMol. The TEM-1 crystal structure also contained one mutation (M182T), which was reverted to the original TEM-1 sequence for the MD simulations. The TEM-116 crystal structure was obtained in complex with imipenem, which was replaced by meropenem. The SFC-1 crystal structure contained a mutation (E166A), which was reverted to wild type based on an S70A meropenem-bound variant (PDB: 4EUZ). The apoenzyme structures of CTX-M-16, TEM-52, KPC-2, NMC-A, and SME-1 were aligned with the SFC-1-meropenem complex to build the respective acyl-enzyme using the meropenem coordinates from the SFC-1 structure.

The AE complex with meropenem in its Δ^2 tautomer was parametrized with RESP charges based on HF/6-31+G(d) density and GAFF parameters (https://doi.org/10.6084/m9.figshare.8158097.v1). The protein was parametrized with the ff12SB forcefield. Apart from the deacylating water, all crystallographic water molecules were deleted. Propka3.1[17] was used to calculate the protonation state, and hydrogens were added using tLeap.[18] Each system was solvated with TIP4P-Ew in a 10 Å box and neutralized by adding chloride or sodium ions. The QM region was treated at the DFTB2 level. The QM region comprised 41 atoms and 3 link atoms (Fig S2a):

covalently bound meropenem, from the CB of Ser70 to the S of meropenem, the sidechain of Glu166 from its CG atom, and the deacylating water.

Restraints were added to the simulations to avoid large conformational changes and unwanted bond-breaking in the QM region.[1,2] A weak harmonic restraint (>2.5 Å, 10 kcal/mol/Å^2 force constant) was applied between the carbonyl oxygen and amine, which formed the β-lactam in the meropenem substrate, to maintain a productive conformation. Likewise, a harmonic restraint (>1.2 Å, 10 kcal/mol/Å^2 force constant) was applied to the carbonyl carbon and oxygen of the acylated serine to prevent bond breakage. Three additional restraints (>1.2 Å, 50 kcal/mol/Å^2 force constant) were applied on the water O-H, hydroxyethyl O-H, and β-lactam amine N-H bonds to prevent proton transfer.

1.2. String method free energy calculations

The adaptive string method,[19] incorporated in the Amber18[18] Sander module, was used to calculate the minimum free energy path. The string method can effectively calculate minimal free energy path in multi-dimensional full energy surfaces by projecting multiple collective variables onto a single reaction coordinate.[19] Two collective variables were chosen to calculate free energy profiles (Fig. 2a). The proton transfer was captured by the distance between the hydrogen of the deacylating water and the carboxylate oxygen of Glu166 (δ_{OH}). The nucleophilic attack was defined by the distance between the deacylating water's oxygen and the meropenem carbonyl carbon (δCO). The string method requires defining the reaction's start and end points for each collective variable. The initial guess file contained two points ([1.80645, 3.52351] and [1.00866, 1.46509]) describing reaction coordinates that are slightly before the AE or after the TI. These points were optimized to ensure that the distribution of each coordinate along the equilibrate string is not biased by the endpoints. The optimization was done by adjusting the endpoints until the equilibrated string would not overshoot the given points. String calculations were performed for 28 string nodes, using the same QM/MM equilibrated starting structure as input for each node. String calculations were run for a total of 500 ps. The string was equilibrated for 25 ps plus an additional 25 ps as

defined in the STOP_STRING file, and the free energy profile was subsequently obtained from sampling for 450 ps (Fig. S2).

Free-energy profiles were used to identify the AC, TS, and TI frames from the string calculation as follows: Because the ground state is comparably broad and undefined, we assigned all frames with reaction coordinates between -0.5 and 2.0 to this state. For the TS and TI, we first located their reaction coordinates for each variant (BlaC, TS: 5.0, TI: 5.6; CTXM, TS: 4.9, TI: 5.5; SHV, TS: 5.0 TI: 5.5; TEM1, TS: 5.7, TI: 5.3; TEM52, TS: 5.0, TI: 5.4; TEM116, TS: 4.8, TI: 5.4; KPC, TS: 4.6, TI: 5.7; NMC, TS: 4.7, TI: 5.8; SFC, TS: 4.7, TI: 5.6; SME, TS: 4.6, TI: 5.5). Subsequently, we assigned all frames ±0.2 from the identified reaction coordinates to the respective TS and TI ensembles.

1.3. 2D umbrella sampling free energy calculations

2D umbrella sampling was used to calculate free energy surfaces and barriers. To explore the free-energy surface, single structures equilibrated through 300 ps QM/MM were submitted to 2D umbrella sampling. Reaction coordinates ranging from 3.5 to 1.4 for the nucleophilic attack and 0.8 to -0.8 for the proton transfer were sampled, resulting in 374 windows. The proton transfer reaction coordinate was defined by the distance between the Glu166 carboxylate O and the deacylating water H minus the distance between the deacylating water H and O. This range was extended compared to our previous work to ensure that the broad AE energy well was sampled exhaustively. Based on the equilibrated structure after 300 ps, umbrella sampling was done for 2 ps on each window to obtain 2D energy surfaces (Fig. 2c+S3, final 2D surfaces show the combined data from this 2 ps sampling and the 100 ps sampling of the minimum energy path described below). All studied β-lactamases have similar minimum free energy paths on full energy surfaces obtained through minimum energy path surface analysis (MEPSA).[20] The final potential of mean force for the two reaction coordinates was obtained through the weighted-histogram analysis method (WHAM).[21] Subsequently, free energy reaction barriers were calculated starting from ten independent configurations 15 ps apart from the 300 ps QM/MM simulations of the AE complex. For some of the picked frames, umbrella sampling did not run to completion due to the misalignment of the deacylating water at the active site in an inappropriate position for

nucleophilic attack and deprotonation. If the umbrella sampling did not run properly, simulations were discarded and restarted with a different frame. 28 windows based on the minimum free energy path along the reaction coordinates were chosen, starting from the reactant structure towards the product structure for more extensive 100 ps umbrella sampling for each window, resulting in the barriers in Fig. 2c.

1.4. Electric field calculations

The electric field projected by the enzyme onto the reactive carbonyl moiety of the β-lactam was calculated using FieldTools (https://github.com/bunzela/FieldTools). FieldTools relies on Coulomb's law using the point charges from the system's topology file and the coordinates from the input trajectory to calculate electric fields along a target bond or at an atom. The electric field vector $\vec{E}$ was calculated from the Coulomb constant k, and the vector $\vec{r}_i$ from the carbonyl carbon (C=O) to all charges Q_i in the system (Eq. S1). Subsequently, the effective field E_{eff} projected along the carbonyl carbon was calculated from the scalar product of the directional unity vector $\vec{d}$ along the carbonyl carbon (C=O) and the total field $\vec{E}$ (Eq. S2). To quantify electric field effects, the analysis presented here focuses on the magnitude of the field vector E_{eff} (Eq. S3).

$$\vec{E} = \sum_i k \frac{Q_i}{\vec{r}_i^{\,2}} \qquad \text{Eq. S1}$$

$$\vec{E}_{eff} = \vec{E} \cdot \vec{d} \qquad \text{Eq. S2}$$

$$E_{eff} = \left|\vec{E}_{eff}\right| \qquad \text{Eq. S3}$$

2. Supporting Tables

Tab. S1 | Free energy barriers for meropenem hydrolysis.

β-lactamase	k_{cat} or k_3 [a] (s^{-1})	$\Delta G^{\ddagger}_{exp}$ (kcal/mol)	$\Delta G^{\ddagger}_{calc}$ String (kcal/mol)	$\Delta G^{\ddagger}_{calc}$ US (kcal/mol)
BlaC	0.0004 [b]	21.5	12.1 ± 0.3	16.4 ± 0.3
CTX-M-16	0.007 [c]	20.5	13.3 ± 0.3	19.0 ± 0.7
SHV-1	0.0017 [d]	21.4	15.1 ± 0.2	17.5 ± 0.3
TEM-1	0.00023 [d]	22.6	16.7 ± 0.2	21.5 ± 0.3
TEM-52	0.0082 [e]	20.4	19.2 ± 0.3	21.3 ± 0.4
TEM-116	0.00023 [d]	22.6	19.1 ± 0.3	23.6 ± 0.9
KPC-2	4.0 [f]	16.7	5.7 ± 0.2	13.8 ± 1.0
NMC-A	12 [g]	16.1	6.0 ± 0.2	12.2 ± 0.7
SFC-1	6.5 [h]	16.4	6.8 ± 0.3	16.6 ± 0.8
SME-1	8.9 [i]	16.3	6.4 ± 0.2	14.8 ± 0.9

[a] Where available, k_3 values corresponding to the deacylation of the AE intermediate are given.
[b] k_3 value from Ref. 3, which also gives rates for acylation (k_2 = 0.019 s^{-1}) and k_{cat} (0.00077 s^{-1}). Note that Ref. 11 gives a comparable k_{cat} value of 0.0013 s^{-1}.
[c] k_{cat} value from Ref. 4 for CTX-M-14, which has a comparable activity spectrum as CTX-M-16 and differs by only 2 mutations (A206V and G214D).
[d] k_{cat} values from Ref. 5. TEM-116 only differs by 2 mutations from TEM-1 (I84V and V184A). These mutations predominantly affect the stability of TEM-1 and not its substrate spectrum; thus, the same k_{cat} value was taken for both enzymes.
[e] k_3 value from Ref. 6, which also gives a rate for acylation (k_2 = 0.00019 s^{-1}).
[f] k_{cat} value corresponding to the average value given Refs. 5,22,23 (3.6 s^{-1}, 3.4 s^{-1}, and 5.0 s^{-1}).
[g] k_{cat} value from Ref. 24.
[h] k_{cat} value from Ref. 25.
[I] k_{cat} value from Ref. 26.

Tab. S2 | Calculated electric fields.

β-lactamase	$\Delta G^{\ddagger}_{exp}$ (kcal/mol)	Electric Field 2D Umbrella Sampling [a] (MV/cm)	Electric Field String Method [a] (MV/cm)
		Total	Total
BlaC	21.5	-105 ± 6	-90 ± 8
CTX-M-16	20.5	-81 ± 5	-88 ± 6
SHV-1	21.4	-98 ± 6	-96 ± 2
TEM-1	22.6	-81 ± 4	-70 ± 3
TEM-52	20.4	-80 ± 6	-77 ± 11
TEM-116	22.6	-53 ± 5	-71 ± 10
KPC-2	16.7	-97 ± 5	-96 ± 4
NMC-A	16.1	-111 ± 6	-107 ± 4
SFC-1	16.4	-98 ± 4	-100 ± 9
SME-1	16.3	-113 ± 6	-101 ± 5
Slope ((MV/cm)/(kcal/mol)) [a]		4.5	3.9

[a] Fields were calculated in the transition state from either string or 2D umbrella sampling.

3. Supporting Figures

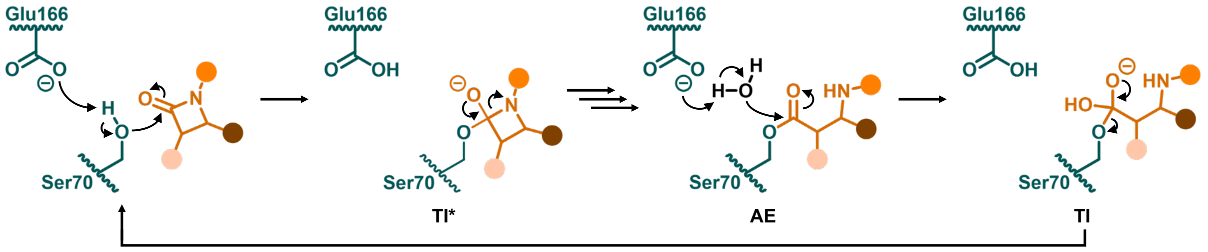

Fig. S1 | Carbapenemase mechanism.

Class A β-lactamases cleave β-lactams (orange) in a two-step mechanism. In the first step, an acyl-enzyme state (AE) is formed *via* a tetrahedral intermediate (TI*). Proton transfer from Glu166 to the β-lactam amine leaving group proceeds via an extended hydrogen-bonding network, probably involving Lys73 and Ser130 (additional steps indicated by a triple arrow).[27] The AE is then hydrolyzed *via* a second tetrahedral intermediate (TI). Our work focuses on the hydrolysis of the AE because this step is rate-limiting in many non-carbapenemases.[3,11] Although some non-carbapenemases, such as TEM-52, are not rate-limited by AE hydrolysis,[6] AE cleavage must typically be substantially accelerated in non-carbapenemases to provide biologically relevant activity. Note that various highly active carbapenemases, such as KPC-2, remain rate-limited by AE cleavage and consequently show burst phase kinetics.[22] Also, k_{cat} values might be limited by non-chemical steps such as substrate binding,[28] which could provide an additional source of error.

In our work, we aimed to understand how electric fields accelerate AE hydrolysis to derive novel insights into the molecular origins of β-lactamase activity. The excellent agreement between the E_{eff} vs. $\Delta G^{\ddagger}_{calc}$ analysis and the PCA suggest that the PCA captures catalytically relevant electrostatic effects (Fig 4b and Fig. S9b). It is stressed that the PCA did not involve any experimental or calculated barriers and that questions regarding the rate-limiting step are thus irrelevant for this analysis.

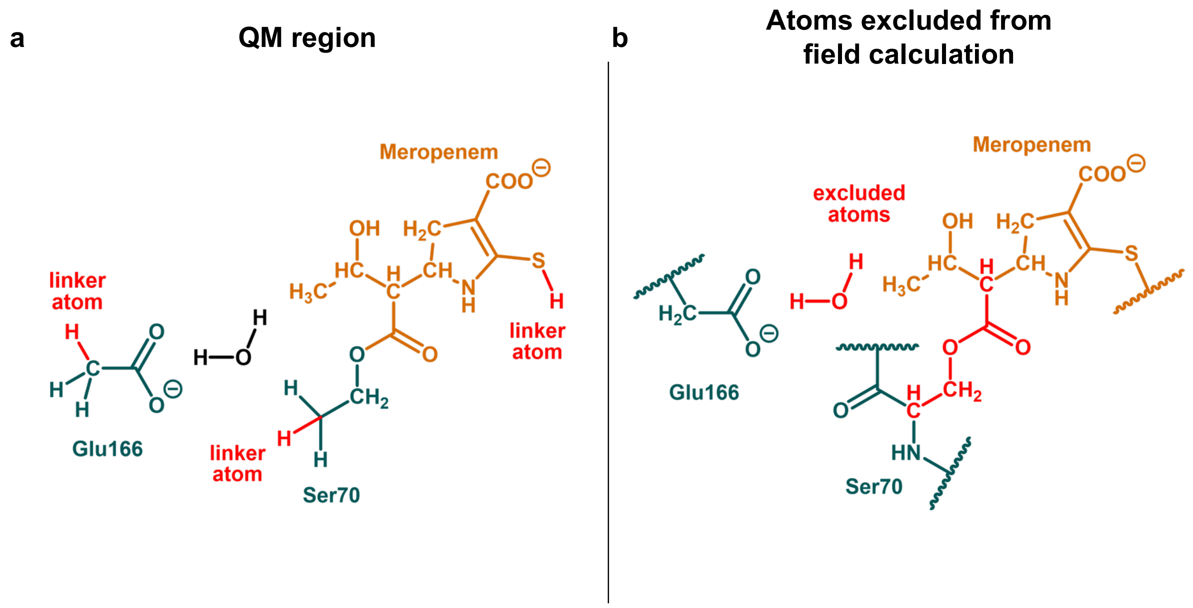

Fig. S2 | QM region and Field calculations.

(a) Atoms in the QM region include parts of the Ser70 and the catalytic Base Glu166 (teal). In addition, the deacylating water (black) and parts of meropenem (orange) are included. Linker atoms are indicated in red. **(b)** During electric field calculation, fields of the atoms neighboring the C=O bound were excluded to mask effects intrinsic to the reaction. Excluded atoms comprise the deacylating water and parts of Ser70 and meropenem (red).

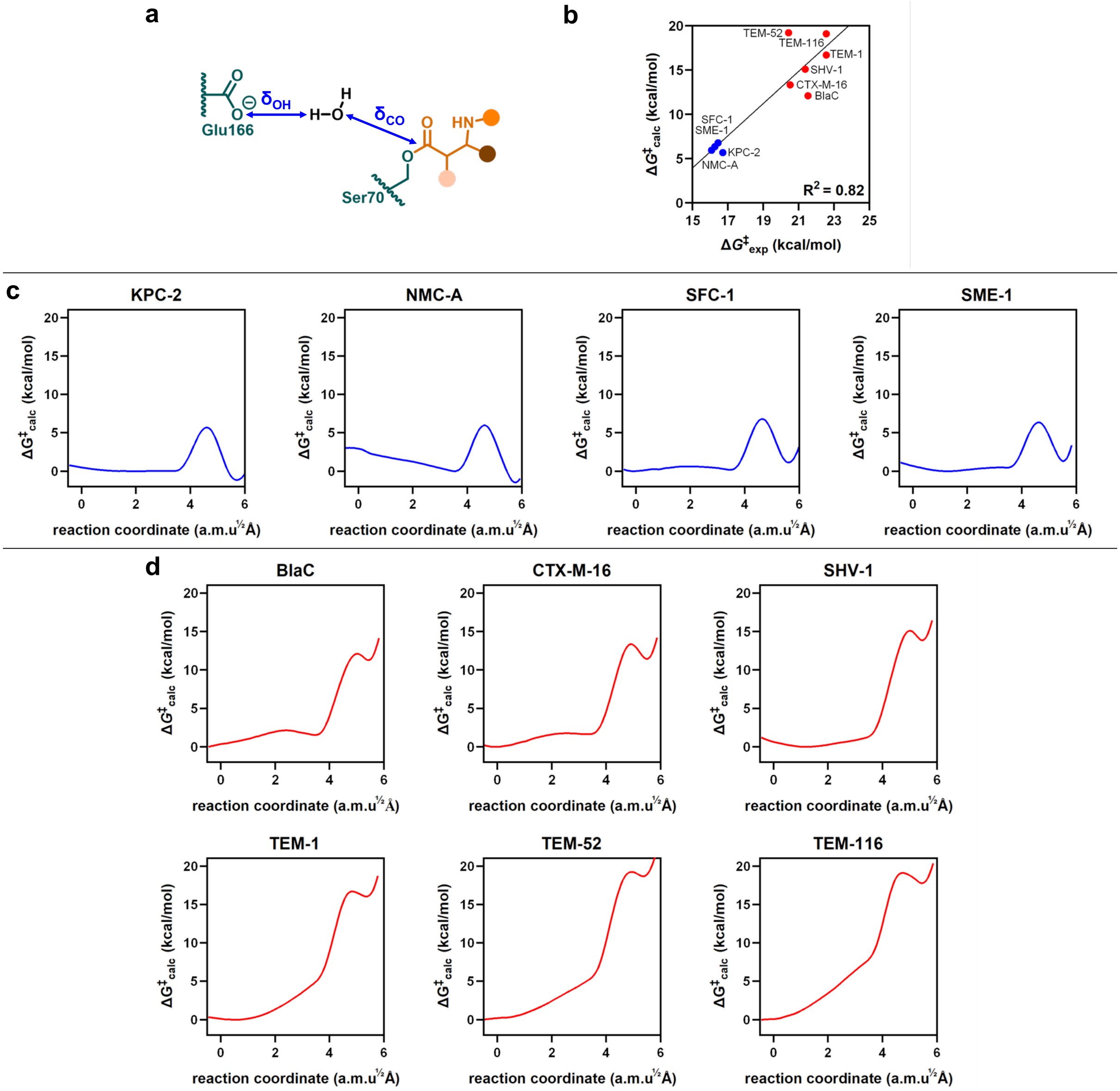

Fig. S3 | Free energy barriers from string calculations.

(a) The δ_{OH} and δ_{CO} distances were sampled during the string calculation. **(b)** Free energies calculated with the String method correlate well with the experimental activation energies (blue: carbapenemases; red: non-carbapenemases). **(c)** Potential mean force (PMF) profiles of carbapenemases measured along minimum free energy path. The highest energy point on each profile represents the transition state (TS) before tetrahedral intermediate (TI) formation. **(d)** PMF profiles of six carbapenem-inhibiting enzymes. Non-carbapenemases show higher deacylation barriers and later transition states than active carbapenemases.

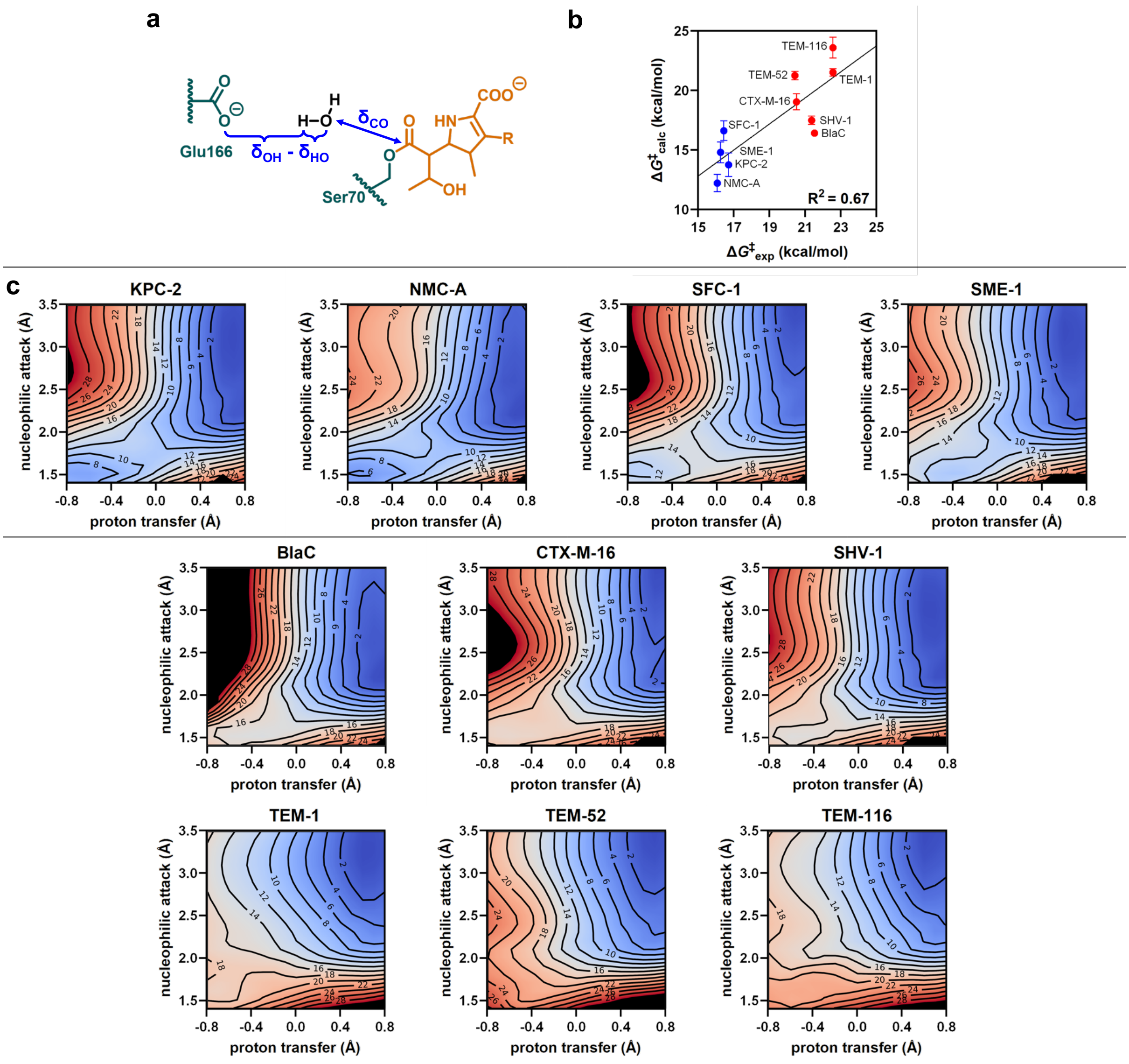

Fig. S4 | Free energy barriers from 2D umbrella sampling.

(a) The sampled reaction coordinates comprise the proton transfer defined by the distance between the Glu166 carboxylate O and the deacylating water H minus the distance between the deacylating water H and O (δ_{OH} - δ_{HO}), and the nucleophilic attack of water onto the AE intermediate (δ_{CO}). **(b)** Free energy barriers calculated from 100 ps 2D umbrella sampling along the minimum free energy path correlate well with the experimental activation energies (blue: carbapenemases; red: non-carbapenemases). **(c)** 2D free energy surfaces of carbapenemases and **(d)** non-carbapenemases were calculated for the deacylation reaction for 2 ps sampling of each window (surfaces represent combined data from the 2 ps calculations and the 100 ps minimum free energy path sampling). Similar to the TS energies from the String calculations (Fig. S2), the difference in TS and TI energies is minor in the non-carbapenemases.

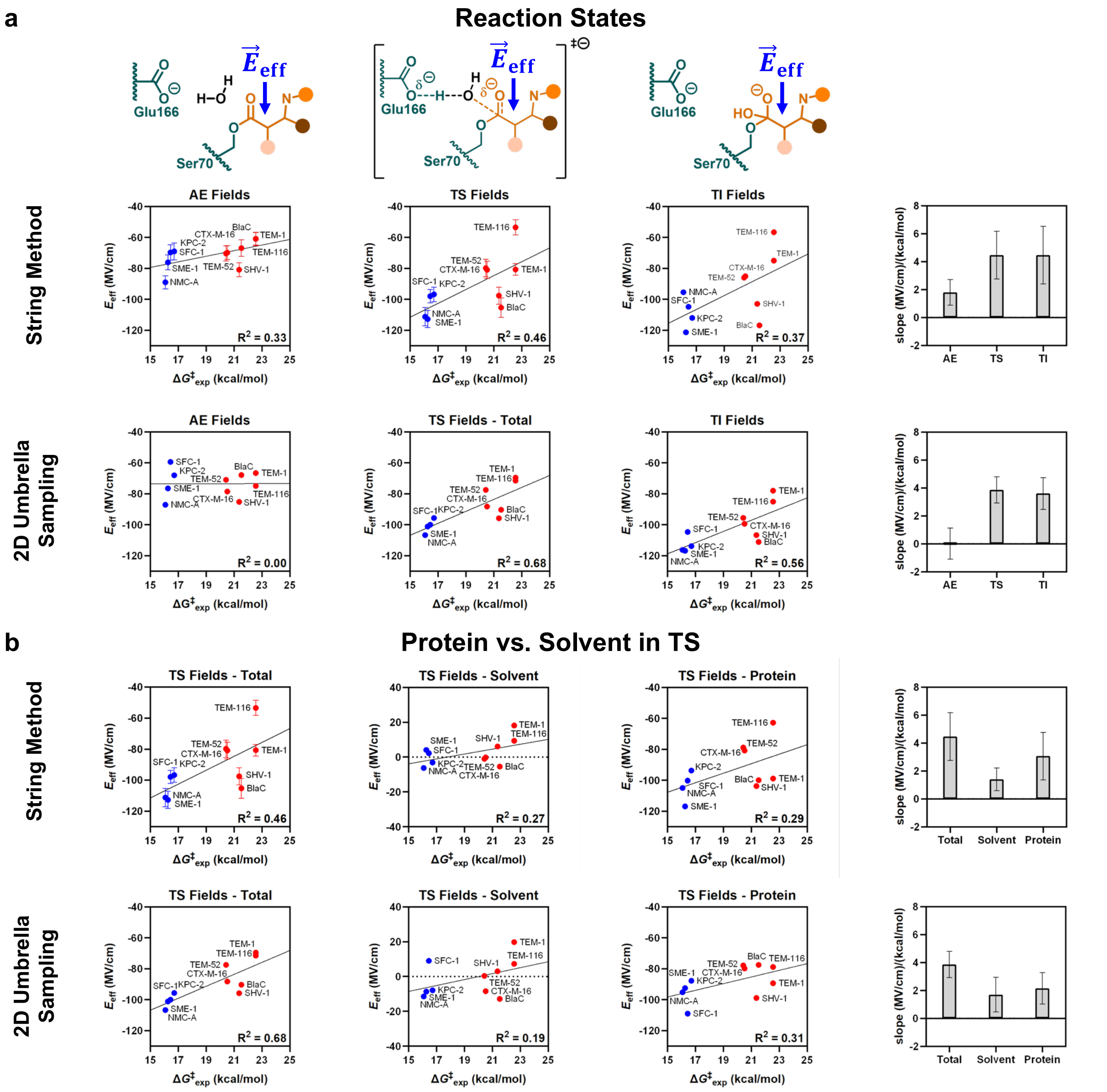

Fig. S5 | Electric fields decomposition into individual states.

(a) Total electric field of the acyl-enzyme (AE), the transition state (TS), and the tetrahedral intermediate (TI) calculated from trajectories based on the string method (top) and 2D umbrella sampling (bottom). Slopes resulting from the E_{eff} vs. $\Delta G^{\ddagger}_{\text{calc}}$ analysis are shown on the right. **(b)** Decomposition of the electric field in the TS into its contributions from solvent and protein shows that the overall effect is dominated by the protein (top: string method; bottom: 2D umbrella sampling, left: slopes resulting from the E_{eff} vs. $\Delta G^{\ddagger}_{\text{calc}}$ analysis.

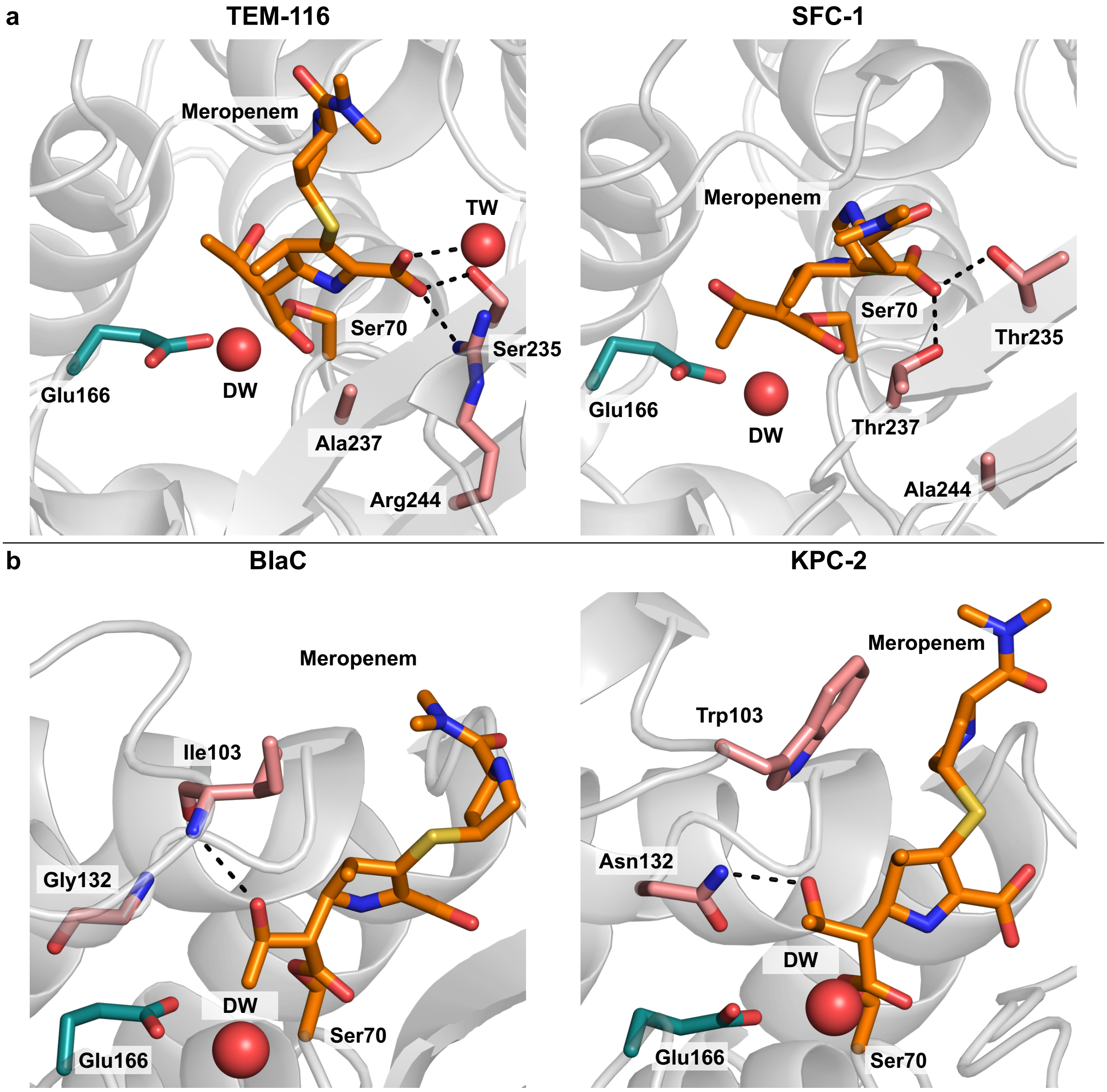

Fig. S6 | Active site interactions in carbapenemases and non-carbapenemases. **(a)** Salt bridge interaction between Arg244 and the meropenem carboxylate in the active site of TEM-116 and SFC-1. **(b)** The Asn132Gly mutation in BlaC removes a hydrogen bonding interaction with the 6-1R-hydroxyethyl group of meropenem, which is present in KPC-2 and the other studied carbapenemases. Removal of that hydrogen bond leads to a new interaction of the 6-1R-hydroxyethyl group with Ile103 in BlaC.

a

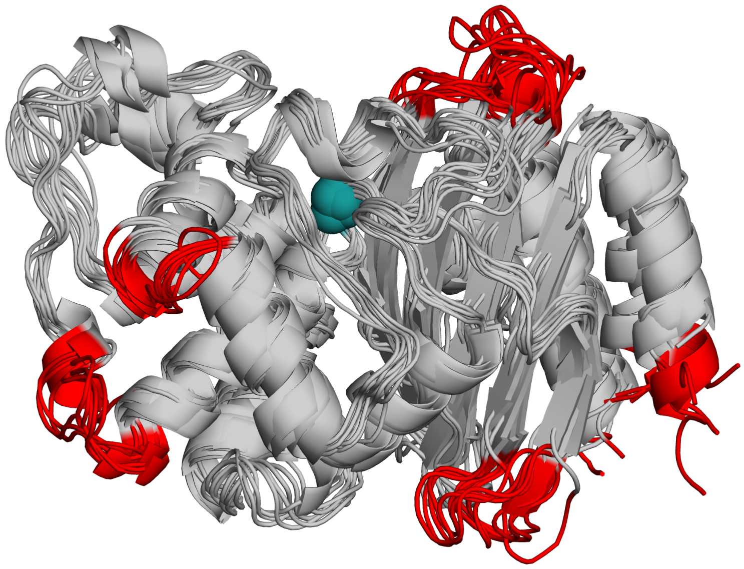

b

```
BlaC      ---- --DLADRFAE LERRYDARLG VYVPATGTTA -AIEYRADER FAFCSTFKAP LVAAVLHQNP LTH--LDKLI TYTSDDIRSI SPVAQQHVQT
CTX-M-16  ---Q TSAVQQKLAA LEKSSGGRLG VALIDTADNT -QVLYRGDER FPMCSTSKVM AAAAVLKQSE TQKQLLNQPV EIKPADLVNY NPIAEKHVNG
SHV-1     ---- SPQPLEQIKL SESQLSGRVG MIEMDLASGR TLTAWRADER FPMMSTFKVV LCGAVLARVD AGDEQLERKI HYRQQDLVDY SPVSEKHLAD
TEM-1     ---- HPETLVKVKD AEDQLGARVG YIELDLNSGK ILESFRPEER FPMMSTFKVL LCGAVLSRVD AGQEQLGRRI HYSQNDLVEY SPVTEKHLTD
TEM-52    ---- HPETLVKVKD AEDQLGARVG YIELDLNSGK ILESFRPEER FPMMSTFKVL LCGAVLSRVD AGQEQLGRRI HYSQNDLVKY SPVTEKHLTD
TEM-116   ---- HPETLVKVKD AEDQLGARVG YIELDLNSGK ILESFRPEER FPMMSTFKVL LCGAVLSRID AGQEQLGRRI HYSQNDLVEY SPVTEKHLTD
KPC-2     ---- ---AEPFAKL EQDFGGSIGV YAMDTGSGA- -TVSYRAEER FPLCSSFKGF LAAAVLARSQ QQAGLLDTPI RYGKNALVPW SPISEKYLTT
NMC-A     ---- NTKGIDEIKN LETDFNGRIG VYALDTGSGK -SFSYRANER FPLCSSFKGF LAAAVLKGSQ DNRLNLNQIV NYNTRSLEFH SPITTKYKDN
SFC-1     ASQP PQVTVDKLKR LENDFGGRIG VYAIDTGSNK -TFGYRANER FPLCSSFKGF LAAAVLSKSQ QQEGLLNQRI RYDNRVMEPH SPVTEKQITT
SME-1     --NK SDAAAKQIKK LEEDFDGRIG VFAIDTGSGN -TFGYRSDER FPLCSSFKGF LAAAVLERVQ QKKLDINQKV KYESRDLEYH SPITTKYKGS

BlaC           GMTIGQLCDA AIRYSDGTAA NLLLADLGGP GGGTAAFTGY LRSLGDTVSR LDAEEPELNR DPPGDERDTT TPHAIALVLQ QLVLGNALPP
CTX-M-16       TMTLAELSAA ALQYSDNTAM NKLIA----Q LGGPGGVTAF ARAIGDETFR LDRTEPTLNT AIPGDPRDTT TPRAMAQTLR QLTLGHALGE
SHV-1          GMTVGELCAA AITMSDNSAA NLLLA----T VGGPAGLTAF LRQIGDNVTR LDRWETELNE ALPGDARDTT TPASMAATLR KLLTSQRLSA
TEM-1          GMTVRELCSA AITMSDNTAA NLLLT----T IGGPKELTAF LHNMGDHVTR LDRWEPELNE AIPNDERDTT MPAAMATTLR KLLTGELLTL
TEM-52         GMTVRELCSA AITMSDNTAA NLLLT----T IGGPKELTAF LHNMGDHVTR LDRWEPELNE AIPNDERDTT TPAAMATTLR KLLTGELLTL
TEM-116        GMTVRELCSA AITMSDNTAA NLLLT----T IGGPKELTAF LHNMGDHVTR LDRWEPELNE AIPNDERDTT MPVAMATTLR KLLTGELLTL
KPC-2          GMTVAELSAA AVQYSDNAAA NLLLK----E LGGPAGLTAF MRSIGDTTFR LDRWELELNS AIPGDARDTS SPRAVTESLQ KLTLGSALAA
NMC-A          GMSLGDMAAA ALQYSDNGAT NIILE---RY IGGPEGMTKF MRSIGDEDFR LDRWELDLNT AIPGDERDTS TPAAVAKSLK TLALGNILSE
SFC-1          GMTVAELSAA TLQYSDNGAA NLLLE---KL IGGPEGMTSF MRSIGDNVFR LDRWELELNS AIPGDDRDTS TPKAVAESMQ KLAFGNVLGL
SME-1          GMTLGDMASA ALQYSDNGAT NIIME---RF LGGPEGMTKF MRSIGDNEFR LDRWELELNT AIPGDKRDTS TPKAVANSLN KLALGNVLNA

BlaC           DKRALLTDWM ARNTTGAKRI RAGFPADWKV IDKTGTGDY- GRANDIAVVW SPTGVPYVVA VMSDRAGGGY DAEPREALLA EAATCVAGVL A
CTX-M-16       TQRAQLVTWL KGNTTGAASI RAGLPTSWTA GDKTGSGGY- GTTNDIAVIW PQGRAPLVLV TYFTQPQQNA ES-RRDVLAS AARIIAEGL- -
SHV-1          RSQRQLLQWM VDDRVAGPLI RSVLPAGWFI ADKTGAGER- GARGIVALLG PNNKAERIVV IYLRDTPASM AE-RNQQIAG IGAALIEHWQ R
TEM-1          ASRQQLIDWM EADKVAGPLL RSALPAGWFI ADKSGAGER- GSRGIIAALG PDGKPSRIVV IYTTGSQATM DE-RNRQIAE IGASLIKHW- -
TEM-52         ASRQQLIDWM EADKVAGPLL RSALPAGWFI ADKSGASER- GSRGIIAALG PDGKPSRIVV IYTTGSQATM DE-RNRQIAE IGASLIKHW- -
TEM-116        ASRQQLIDWM EADKVAGPLL RSALPAGWFI ADKSGAGER- GSRGIIAALG PDGKPSRIVV IYTTGSQATM DE-RNRQIAE IGASLIKHW- -
KPC-2          PQRQQFVDWL KGNTTGNHRI RAAVPADWAV GDKTGTCGVY GTANDYAVVW PTGRAPIVLA VYTRAPNKDD KH-SEAVIAA AARLALEGLG V
NMC-A          HEKETYQTWL KGNTTGAARI RASVPSDWVV GDKTGSCGAY GTANDYAVVW PKNRAPLIIS VYTTKNEKEA KH-EDKVIAE ASRIAIDNLK -
SFC-1          TERHQLMDWF KGNTTGGARI RASVPANWVV GDKTGTCGVY GTANDYAVIW PVAHAPIVLA VYTSKPDRNS KH-SDAVIAD ASRIVLESFN I
SME-1          KVKAIYQNWL KGNTTGDARI RASVPADWVV GDKTGSCGAY GTANDYAVIW PKNRAPLIVS IYTTRKSKDD KH-SDKTIAE ASRIAIQAID -
```

Fig. S7 | Structure-based sequence alignment.

(a) Overlay of all studied enzymes for structure-based sequence alignment. Loops that were excluded from the alignment and per-residue analysis due to their varying lengths between variants are shown in red. All loops are away from the active site (catalytic serine: teal spheres). **(b)** The excluded residues are highlighted in red in the multiple sequence alignment.

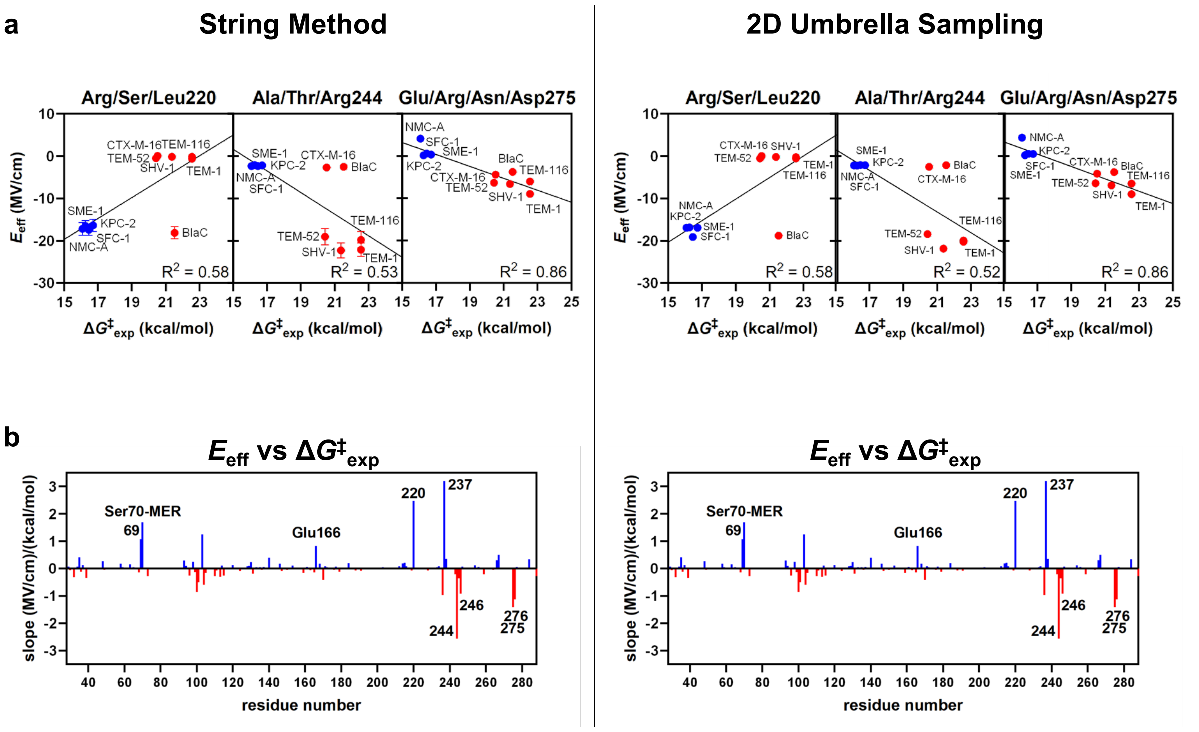

Fig. S8 | Per-residue electric fields.

The FieldTools script readily allows the decomposition of the total electric field into individual contributions (String Method: left, 2D Umbrella sampling: right). (**a**) The E_{eff} of several residues substantially changed with activity, as shown by the (**b**) slopes determined from E_{eff} vs. $\Delta G^{\ddagger}_{\text{calc}}$. These residues comprised obvious contributors, such as the oxyanion-hole donating residue 237 and non-obvious residues up to 15 Å away from the oxyanion (Ser70-MER: Field effect of the meropenem acylated Ser70).

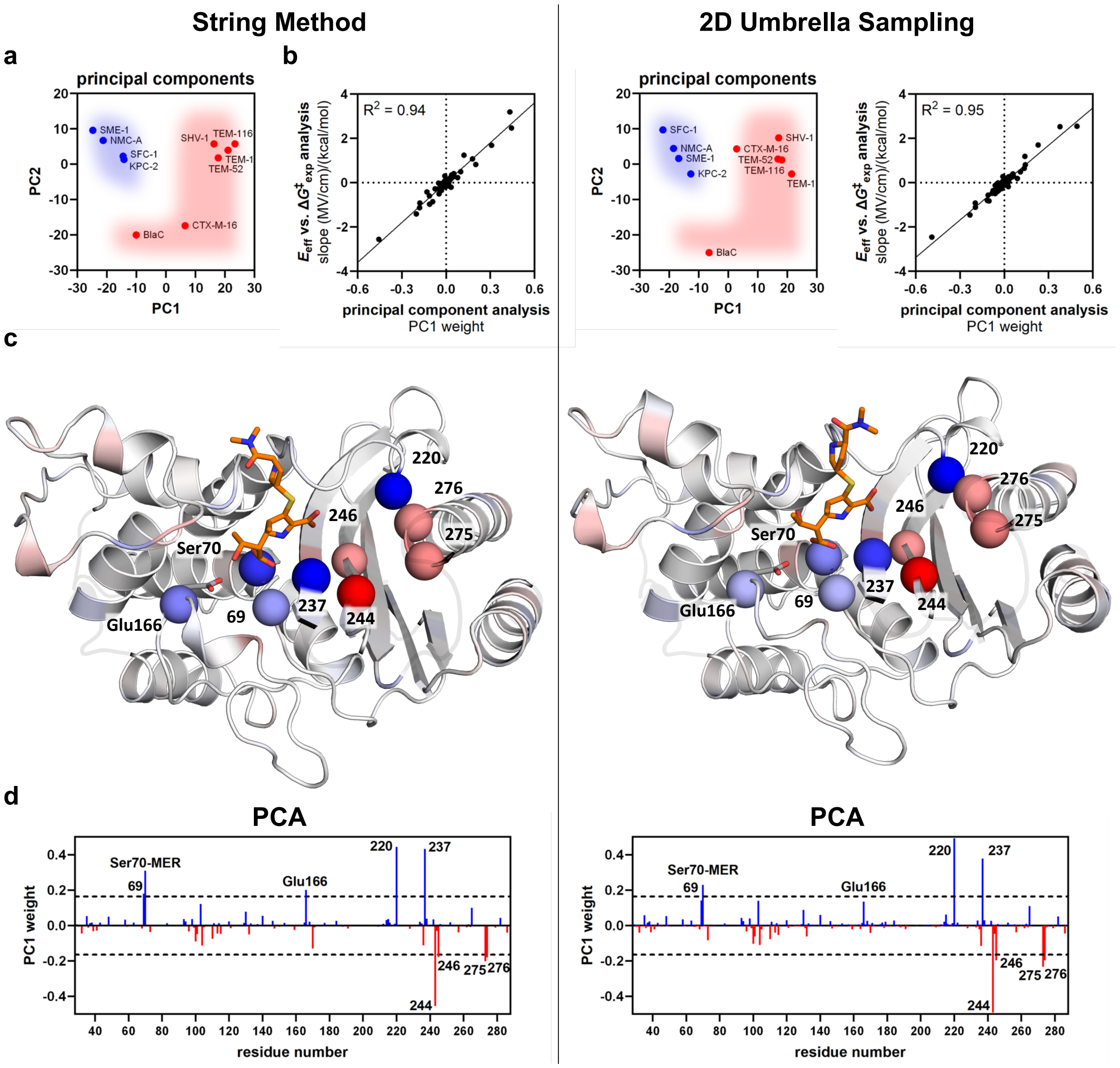

Fig. S9 | PCA reveals residues key to electrostatic catalysis.

Results from the string calculations **(left)** agree well with those from 2D umbrella sampling **(right)**. **(a)** PCA of the per-residue E_{eff} clearly distinguishes carbapenemases (blue) from non-carbapenemases (red, shaded areas added for illustration only). **(b)** Per-residue slopes from the E_{eff} vs. $\Delta G^{\ddagger}_{calc}$ analysis agree well with the PC1 weights, suggesting that the PCA captures catalytically relevant field effects. **(c)** PC1 weights of each residue projected onto the β-lactamase scaffold reveals that a cluster of seven residues plus Ser70 and Glu166 (spheres) dominates E_{eff} (blue: beneficial; red: detrimental, meropenem: orange sticks). **(c)** Electrostatic contribution of each protein residue suggested by PC1 weights. Critical residues were identified as residues whose field varied by more than 2.5 standard deviation cutoff from all PC1 values (blue: beneficial; red: detrimental; Ser70-MER: Field effect of the meropenem acylated Ser70).

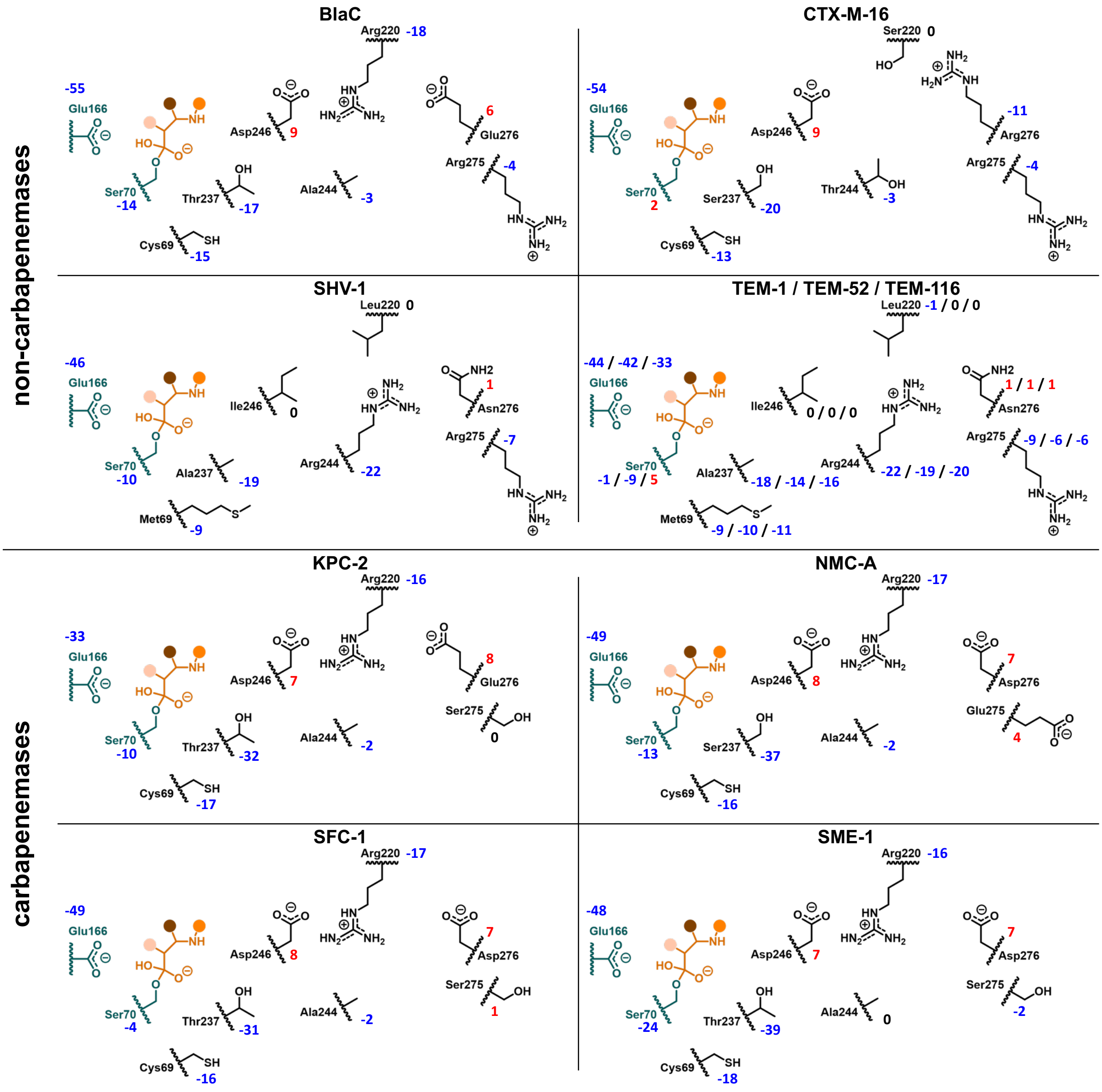

Fig. S10 | Residues distinguishing carbapenemases from non-carbapenemases. Critical residues were identified by PCA in the non-carbapenemases (top) and carbapenemases (bottom). These residues comprised obvious contributors, such as the oxyanion-hole donating residue 237 and non-obvious residues up to 15 Å away from the oxyanion. E_{eff} values in MV/cm are shown next to each residue. (blue: beneficial; red: detrimental; black: neutral; E_{eff} of Ser70 reflects the combined effect of Ser70 and the covalently-bound meropenem; E_{eff} in the TS from string calculations).

4. References

1. Chudyk, E. I. *et al.* QM/MM simulations as an assay for carbapenemase activity in class A β-lactamases. *Chem Commun* **50**, 14736–14739 (2014).
2. Hirvonen, V. H. A. *et al.* An Efficient Computational Assay for β-Lactam Antibiotic Breakdown by Class A β-Lactamases. *J. Chem. Inf. Model.* **59**, 3365–3369 (2019).
3. Chow, C., Xu, H. & Blanchard, J. S. Kinetic Characterization of Hydrolysis of Nitrocefin, Cefoxitin, and Meropenem by β-Lactamase from Mycobacterium tuberculosis. *Biochemistry* **52**, 4097–4104 (2013).
4. Ishii, Y., Galleni, M., Ma, L., Frère, J.-M. & Yamaguchi, K. Biochemical characterisation of the CTX-M-14 β-lactamase. *Int. J. Antimicrob. Agents* **29**, 159–164 (2007).
5. Queenan Anne Marie, Shang Wenchi, Flamm Robert, & Bush Karen. Hydrolysis and Inhibition Profiles of β-Lactamases from Molecular Classes A to D with Doripenem, Imipenem, and Meropenem. *Antimicrob. Agents Chemother.* **54**, 565–569 (2010).
6. Franceschini, N. *et al.* Meropenem stability to β-lactamase hydrolysis and comparative in vitro activity against several β-lactamase-producing Gram-negative strains. *J. Antimicrob. Chemother.* **49**, 395–398 (2002).
7. Ke, W., Bethel, C. R., Thomson, J. M., Bonomo, R. A. & van den Akker, F. Crystal Structure of KPC-2: Insights into Carbapenemase Activity in Class A β-Lactamases,. *Biochemistry* **46**, 5732–5740 (2007).
8. Swarén, P. *et al.* X-ray Analysis of the NMC-A β-Lactamase at 1.64-Å Resolution, a Class A Carbapenemase with Broad Substrate Specificity. *J. Biol. Chem.* **273**, 26714–26721 (1998).
9. Fonseca, F. *et al.* The Basis for Carbapenem Hydrolysis by Class A β-Lactamases: A Combined Investigation using Crystallography and Simulations. *J. Am. Chem. Soc.* **134**, 18275–18285 (2012).
10. Sougakoff, W. *et al.* Structure of the imipenem-hydrolyzing class A β-lactamase SME-1 from ıt Serratia marcescens. *Acta Crystallogr. Sect. D* **58**, 267–274 (2002).
11. Hugonnet, J.-E., Tremblay, L. W., Boshoff, H. I., Barry, C. E. & Blanchard, J. S. Meropenem-Clavulanate Is Effective Against Extensively Drug-Resistant Mycobacterium tuberculosis. *Science* **323**, 1215–1218 (2009).
12. Chen, Y., Delmas, J., Sirot, J., Shoichet, B. & Bonnet, R. Atomic Resolution Structures of CTX-M β-Lactamases: Extended Spectrum Activities from Increased Mobility and Decreased Stability. *J. Mol. Biol.* **348**, 349–362 (2005).
13. Nukaga, M. *et al.* Inhibition of Class A β-Lactamases by Carbapenems: Crystallographic Observation of Two Conformations of Meropenem in SHV-1. *J. Am. Chem. Soc.* **130**, 12656–12662 (2008).
14. Minasov, G., Wang, X. & Shoichet, B. K. An Ultrahigh Resolution Structure of TEM-1 β-Lactamase Suggests a Role for Glu166 as the General Base in Acylation. *J. Am. Chem. Soc.* **124**, 5333–5340 (2002).
15. Orencia, M. C., Yoon, J. S., Ness, J. E., Stemmer, W. P. C. & Stevens, R. C. Predicting the emergence of antibiotic resistance by directed evolution and structural analysis. *Nat. Struct. Biol.* **8**, 238–242 (2001).

16. Maveyraud, L. *et al.* Structural Basis for Clinical Longevity of Carbapenem Antibiotics in the Face of Challenge by the Common Class A β-Lactamases from the Antibiotic-Resistant Bacteria. *J. Am. Chem. Soc.* **120**, 9748–9752 (1998).
17. Olsson, M. H. M., Søndergaard, C. R., Rostkowski, M. & Jensen, J. H. PROPKA3: Consistent Treatment of Internal and Surface Residues in Empirical pKa Predictions. *J. Chem. Theory Comput.* **7**, 525–537 (2011).
18. Case, D. A. *et al.* Amber2018. University of California (2018).
19. Zinovjev, K. & Tuñón, I. Adaptive Finite Temperature String Method in Collective Variables. *J. Phys. Chem. A* **121**, 9764–9772 (2017).
20. Marcos-Alcalde, I., Setoain, J., Mendieta-Moreno, J. I., Mendieta, J. & Gómez-Puertas, P. MEPSA: minimum energy pathway analysis for energy landscapes. *Bioinformatics* **31**, 3853–3855 (2015).
21. Kumar, S., Rosenberg, J. M., Bouzida, D., Swendsen, R. H. & Kollman, P. A. THE weighted histogram analysis method for free-energy calculations on biomolecules. I. The method. *J. Comput. Chem.* **13**, 1011–1021 (1992).
22. Mehta, S. C. *et al.* KPC-2 β-lactamase enables carbapenem antibiotic resistance through fast deacylation of the covalent intermediate. *J. Biol. Chem.* **296**, 100155 (2021).
23. Barnes Melissa D. *et al.* Nacubactam Enhances Meropenem Activity against Carbapenem-Resistant Klebsiella pneumoniae Producing KPC. *Antimicrob. Agents Chemother.* **63**, 10.1128/aac.00432-19 (2019).
24. Mariotte-Boyer, S., Nicolas-Chanoine, M. H. & Labia, R. A kinetic study of NMC-A β-lactamase, an Ambler class A carbapenemase also hydrolyzing cephamycins. *FEMS Microbiol. Lett.* **143**, 29–33 (1996).
25. Fonseca Fátima *et al.* Biochemical Characterization of SFC-1, a Class A Carbapenem-Hydrolyzing β-Lactamase. *Antimicrob. Agents Chemother.* **51**, 4512–4514 (2007).
26. Queenan Anne Marie *et al.* SME-Type Carbapenem-Hydrolyzing Class A β-Lactamases from Geographically Diverse Serratia marcescens Strains. *Antimicrob. Agents Chemother.* **44**, 3035–3039 (2000).
27. Hermann, J. C., Hensen, C., Ridder, L., Mulholland, A. J. & Höltje, H.-D. Mechanisms of Antibiotic Resistance: QM/MM Modeling of the Acylation Reaction of a Class A β-Lactamase with Benzylpenicillin. *J. Am. Chem. Soc.* **127**, 4454–4465 (2005).
28. Fröhlich, C. *et al.* Epistasis arises from shifting the rate-limiting step during enzyme evolution of a β-lactamase. *Nat. Catal.* **in press**, https://doi.org/10.1038/s41929-024-01117-4 (2024).
